# The Detective Value of Magnetically Controlled Robotic Capsule Endoscopy in Patients With Suspected Small Intestinal Disease

**DOI:** 10.3389/fmed.2021.610563

**Published:** 2021-05-25

**Authors:** Xiao-Yu Chen, Wei Da, Rui Liang, Hui-Ning Fan, You-Cai Yi, Ming Chen, Huang-Wen Qin, Jing Zhang, Jin-Shui Zhu

**Affiliations:** Department of Gastroenterology, Shanghai Jiao Tong University Affiliated Sixth People's Hospital, Shanghai, China

**Keywords:** abdominal pain, capsule endoscopy, detective value, enteritis, intestinal disease

## Abstract

**Objective:** To explore the detective value of magnetically controlled robotic capsule endoscopy (MCRCE) in patients with suspected small intestinal disease.

**Patients and Methods:** In total, 1,802 patients with suspected small intestinal disease and negative gastroenteroscopy from Shanghai Jiao Tong University Affiliated Sixth People's Hospital were examined with MCRCE, and the data were collected for further analysis.

**Results:** Among the 1,802 patients who were examined with MCRCE, 974 were diagnosed with small intestinal disease, reaching a positive detection rate of 54.1%. The five most common conditions that were detected include non-specific enteritis in 722 cases (40.1%), small intestinal ulcers in 87 cases (4.8%), abnormal small bowel evacuation in 45 cases (2.5%), small intestinal bleeding in 33 cases (1.8%), and small intestinal yellow spots in 31 cases (1.7%). The running time of the capsules in the small intestine ranged from 85–437 min, with an average of 210.24 ± 89.08 min. No complications, such as intestinal obstruction or capsule retention, were observed in all patients.

**Conclusion:** MCRCE is a safe and non-invasive endoscopic examination with a highly accurate detection rate for small intestinal diseases.

## Introduction

With a total length of 5–7 m in adults, the small intestine is the longest part of the alimentary canal. Diseases of the small intestine are important components of digestive tract diseases. These diseases are clinically present as gastrointestinal bleeding, abdominal pain, diarrhea, and constipation. Lesions of the small intestine mainly consist of benign ulcers, tumors (such as small intestinal adenocarcinoma, lymphoma, and gastrointestinal stromal tumors), chronic inflammation, vascular malformations, polyp, and diverticula (1–3). The incidence of small bowel disease in China is unclear due to insufficient national epidemiological investigations. Besides, the unique structure of the intestine, including its deep position, dissociation from the peritoneum, and multiple composite mesenteries, contributes to the difficulty in diagnosing small intestinal diseases. Therefore, it is urgent to develop a new and effective detective tool for the diagnosis of small intestinal diseases.

Previous studies showed that the detection methods for gastroenterologic diseases include computerized tomography (CT), enterography, digital subtraction angiography, and balloon-assisted enteroscopy. However, these diagnostic methods cannot be widely used for the small intestine because of their low sensitivity, complexity of operation, and invasiveness (4, 5). Moreover, most patients with small bowel diseases cannot tolerate the invasiveness of enteroscopy. Therefore, developing an effective new and non-invasive technology for small bowel diagnosis is an urgent problem that needs to be solved.

The second generation of magnetically controlled robotic capsule endoscopy (MCRCE) is a new and advanced clinical detection technology in China with a high detection rate for small lesions in the small intestinal tract using a non-invasive and painless procedure (3). In this study, MCRCE was utilized to examine patients suspected of having small intestinal diseases in our hospital, and we aimed to explore the detective value and safety profile of MCRCE.

## Materials and Methods

### Study Participants

**Figure 1:**
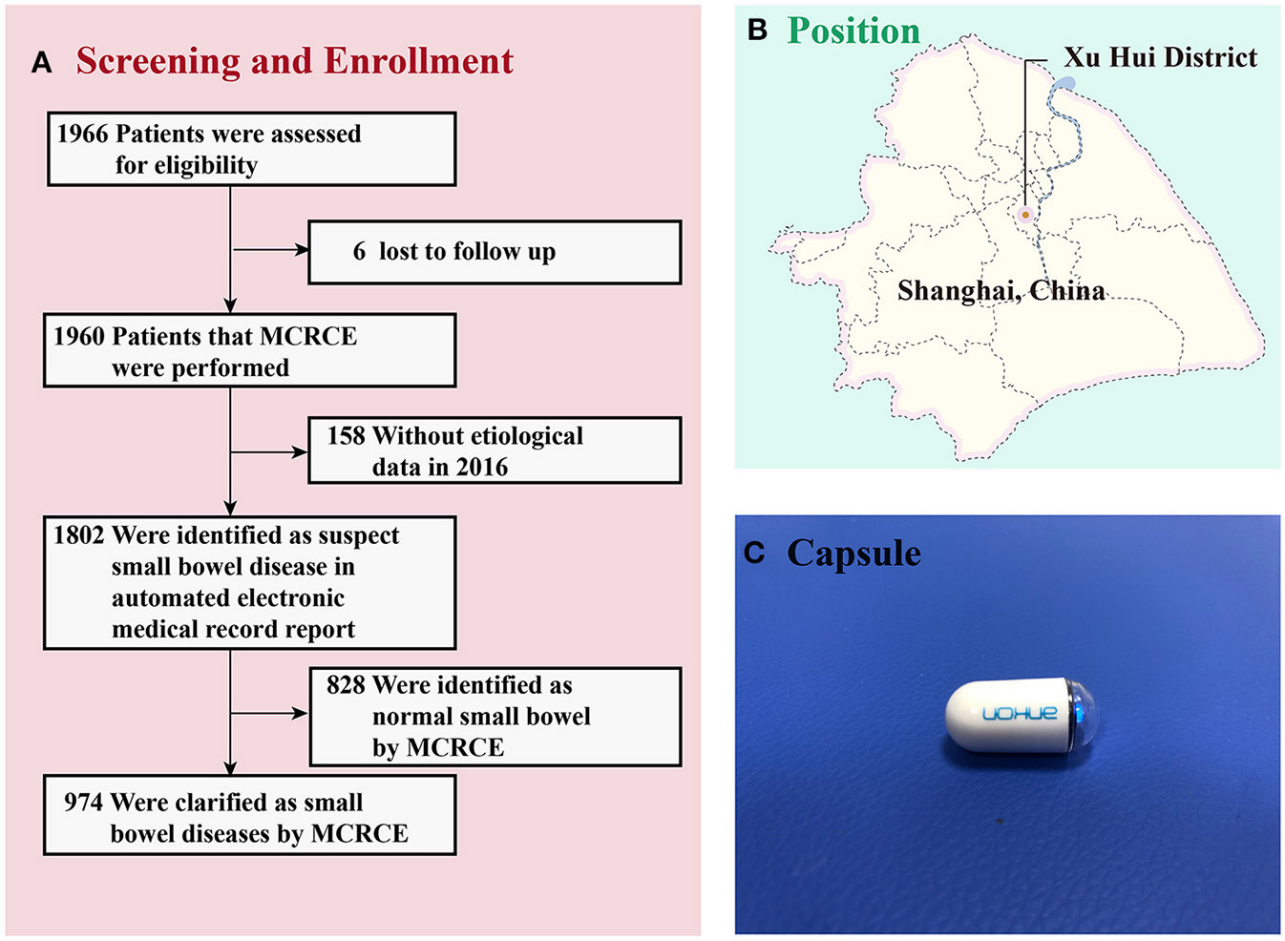
**(A)** Screening and enrollment of patients in the study. **(B)** The place of recruitment in Shanghai (Shanghai Jiao Tong University Affiliated Sixth People's Hospital). **(C)** The NaviCam Magnetically Controlled Capsule Endoscopy from Ankon Medical Technology Co., Ltd. MCRCE, magnetically controlled robotic capsule endoscopy.

Patients from the gastroenterology outpatient and inpatient department of Shanghai Jiao Tong University Affiliated Sixth People's Hospital, who were suspected of having small bowel disease, between March 2016 and May 2019 were included in this study (Figures 1A,B). All patients signed an informed consent form. According to the 2014 China guidelines for the clinical application of capsule endoscopy (6), the inclusion criteria for patients to undergo MCRCE are as follows: (i) presence of chronic abdominal symptoms, such as pain, abdominal distention, and diarrhea; (ii) absence of an organic lesion on gastroscopy and colonoscopy; and (iii) no gastrointestinal obstruction observed on CT imaging. None of the eligible patients used medications (such as proton pump inhibitors, aspirin, non-steroidal anti-inflammatory drugs, or antibiotics) within 2 weeks prior to the MCRCE or ever received preoperative chemotherapy with radiotherapy. Conversely, the following patients were excluded from the study: (i) patients with known or suspected gastrointestinal tract obstruction, stenosis, or fistula; (ii) patients without surgical conditions or that did not agree to any surgical or invasive procedures due to the complication of MCRCE; (iii) patients with dysphagia; (iv) patients with cardiac pacemakers or other implanted electronic instruments; and (v) pregnant women. This clinical research was approved by the Ethics Committee of the Shanghai Jiao Tong University Affiliated Sixth People's Hospital.

### Instrument

MCRCE was developed by Shanghai Ankon Medical Technology Co., Ltd. and Wuhan Ankon Photoelectric Technology Co., Ltd. (State Food Drug Administration, No. 3220109). The NaviCam MCRCE (Ankon, China) comprises a capsule control system, a capsule endoscope, a portable recorder, a capsule positioner, and a display software. The control system includes the testbed, magnetic head, rotary table, and control table. The main specifications of the NaviCam magnetic capsule endoscopy are as follows: size of 27.0 × 11.8 mm, visual angle of 140 ± 14 degrees, shooting frame rate in the stomach or small bowel mode of two pictures per second, working time of 10 14 h, and net weight of 2 g (Figure 1C). The battery life of the capsule was more than 8 h (3).

### Procedural Technique

**Figure 2:**
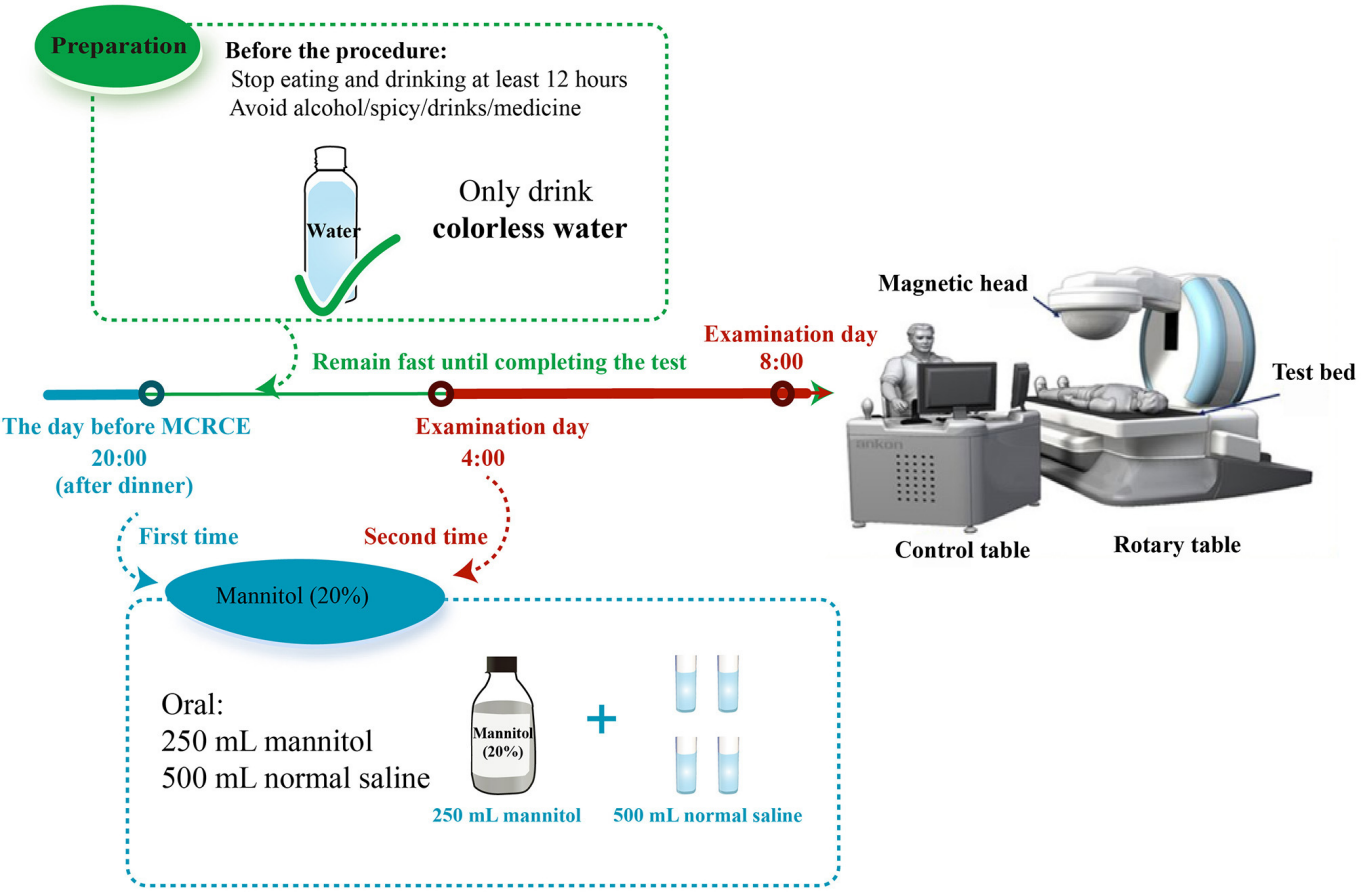
Magnetically controlled robotic capsule endoscopy procedure.

As shown in Figure 2, patients were placed on a slag-free semifluid diet 1 day before the procedure. In addition, 250 ml of 20% mannitol and 500 ml of normal saline solution were administered orally to the patients at 8 p.m. 1 day prior and at 4 a.m. on the day of the procedure. The standard preparation adopted for the gastrointestinal tract is to achieve clear liquid stools with little or no fecal residue. On the examination day, patients had to fast but could drink water. Hungry patients without diabetes could drink water with syrup. Patients were required to take lidocaine hydrochloride mucilage and warm water (250 ml) orally 30–60 min before the examination. Additional warm water (500–800 ml) was administered to patients until a sense of fullness was attained 10 min before the examination. The patients were asked to wear checked clothes (a special clothing that should be worn while undergoing the examination) and lie on the examination bed in a left-lateral position. Afterward, we opened the capsule and waited for approximately 2 min until the voltage of the capsule was stable (over 2,900 mV). Subsequently, we asked the patient to take a sip of water to swallow the capsule while in the left-lateral position. We examined various parts of the esophageal and gastric mucosa by operating the magnetic control handle and changing the patient's position. The examination continued for 15–30 min before transitioning to the small bowel examination mode. The patients were asked to take only colorless liquid within 4 h and consume a regular diet 4 h after the examination. Similarly, patients were advised to avoid walking near strong magnetic fields. The examination was complete when the battery indicator on the checked clothes shut down. The checked clothes were subsequently returned to the doctor the following day. We exported the image data from the portable recorder to the ESnavi software 2.0 (TEAC, China) for interpretation and diagnosis. After the procedure, the patients were asked to pay attention to the discharge of the capsule. The patients were followed up 2 weeks until the capsule was discharged without any discomfort.

### Data Analysis

Images were reviewed independently by two experienced senior physicians who agreed upon the final diagnosis. Non-specific enteritis was defined as an inflammation located in any one of the duodenal, jejunal, and/or ileal parts of the small bowel. Inflammation was defined as having the following manifestations: (i) mucosal lesions (including erosions); (ii) mucosal changes, such as edema and prominent mucosal folds; (iii) changes in the villi, such as flat mucosa and coarsened villi; and (iv) lymphangiectasias/lymphocellular infiltrates (3, 7). The running time of the capsules in the small intestine was recorded and calculated. The positive lesions in the small intestine were identified by MCRCE, and the detection rate of small intestinal disease was recorded.

### Statistical Analysis

Statistical analysis was performed using SPSS 22.0 (IBM, SPSS, Chicago, IL, USA). Data that were normally distributed are expressed as mean ± standard deviation, and other recorded data were expressed in terms of the number of cases and percentages.

## Results

### Participants

In total, 1,802 patients suspected of having intestinal disease were enrolled in the study. These included 811 men and 991 women, aged 14–95 years, with a mean age of 50.10 ± 16.26 years. Abdominal pain and discomfort were observed in 630 (35%) and 290 cases (16.1%), respectively. Other clinical symptoms of the patients included in the study are presented in Table 1.

**Table 1:** Patient demographics and clinical characteristics.

**Characteristic**	**Value**
**Mean of age (range)—yr**	50.1 (14–95)
**Distribution of age—no. (%)**
<20	29 (1.6)
20–30	142 (7.9)
30–40	403 (22.4)
40–50	330 (18.3)
50–60	305 (16.9)
60–70	378 (21)
70–80	147 (8.2)
>80	68 (3.8)
**Male sex—no. (%)**	811 (45.0)
**Small bowel diseases—no. (%)**	974 (54.1)
Non-specific enteritis	722 (40.1)
Ulcer of small intestine	87 (4.8)
Abnormal small bowel evacuation	45 (2.5)
Small bowel bleeding or remote hemorrhage	33 (1.8)
Yellow spots	31 (1.7)
Polyp of small intestine	22 (1.2)
Small intestinal mass	15 (0.8)
Diverticulum of small intestine	8 (0.4)
Absence of villi	5 (0.3)
Vasculopathy	4 (0.2)
Small bowel erythema	1 (0.1)
Pale intestinal mucosa	1 (0.1)
**Symptoms—no. (%)**
Abdominal pain	630 (35.0)
Abdominal discomfort	290 (16.1)
Diarrhea	165 (9.2)
GIB (visible/occult)	149 (8.3)
Abdominal distention	96 (5.3)
Others (Vomiting and others.)	472 (26.2)

*GIB, gastrointestinal bleeding*.

### Safety and Tolerability

During the study's timeline, 1,960 of the 1,966 patients (99.69%) successfully completed the examination. Six of the subjects were lost to follow up. Among the 1,960 participants, 158 were excluded due to the lack of etiological data, while 1,802 were suspected of having small bowel disease. Among these 1,802 participants, 828 had normal findings in their small intestines, while the remaining 974 were identified to have small bowel disease (Figure 1A). All patients had good compliance and successfully swallowed the capsule without any apparent discomfort. All patients excreted the capsule 1–7 days after the examination, and no cases of intestinal obstruction or capsule retention were recorded.

### Magnetic Control Capsule Endoscopy Examination

The operator controlled the capsule into the duodenum in 255 cases (14.2%), while it was naturally transported into the duodenum in the remaining 1,547 cases (85.9%). The image quality and clarity were ideal in most subjects; however, observation of some areas of the small intestinal mucosa is limited by bile and other small bowel contents and secretions. The running time of the capsules in the small intestine was 85–437 min, with an average of 210.24 ± 89.08 min.

### Examination Results

**Figure 3:**
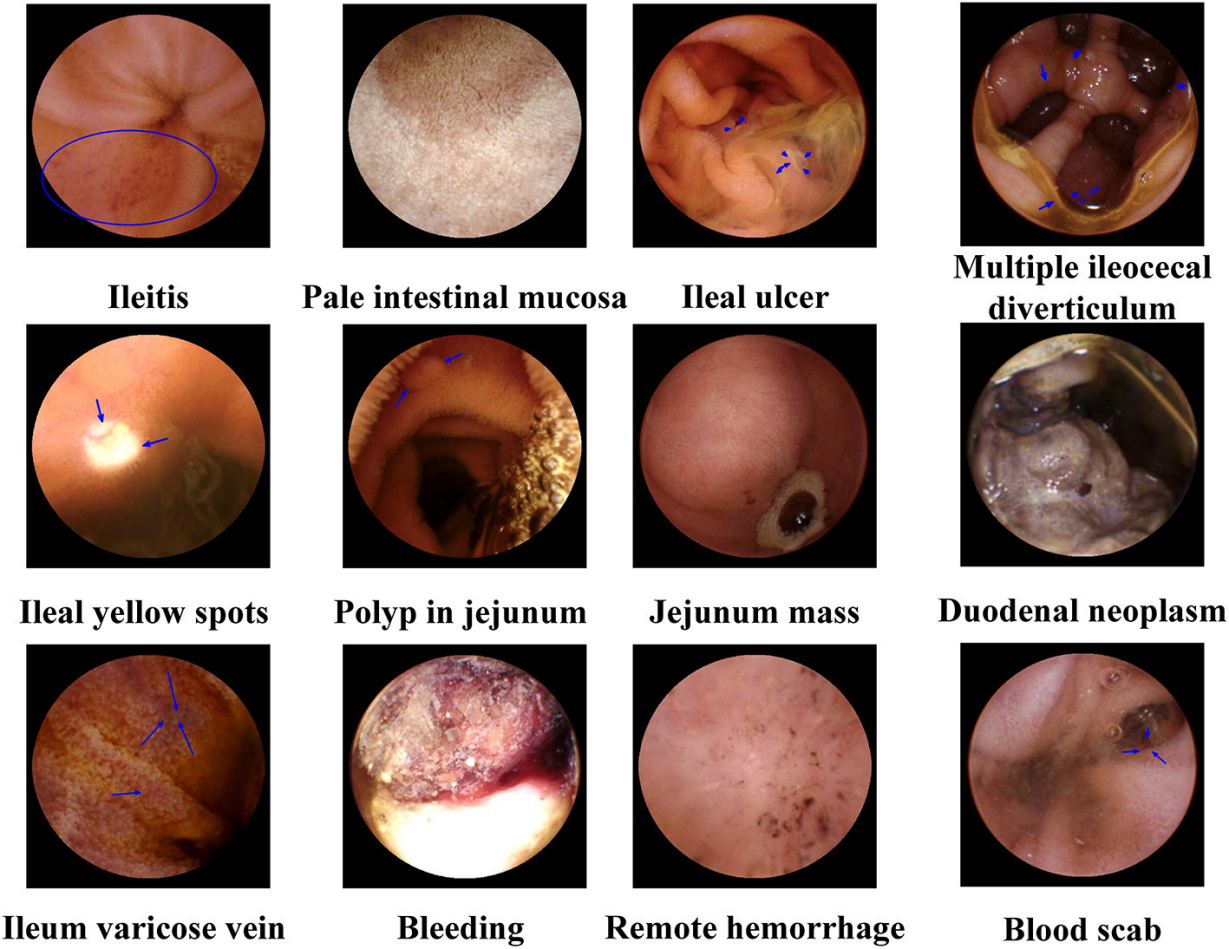
Representative magnetically controlled robotic capsule endoscopy (MCRCE) images of small bowel (duodenum, jejunum, and ileum) lesions.

The MCRCE procedure could detect various small bowel conditions in a total of 974 participants, the summary of which is presented in Tables 1, 2 and Figure 3. Most cases that were detected include non-specific enteritis (722 cases) and small intestinal ulcers (87 cases).

**Table 2:** Types of intestinal diseases detected by MCRCE.

**Small bowel abnormalities**	**Symptoms**						
	**Abdominal pain**	**Abdominal discomfort**	**Diarrhea**	**GIB (visible/occult)**	**Abdominal distention**	**Others**	**Summary**
Non-specific enteritis	320	94	91	35	27	155	722
Ulcer of small intestine	32	15	8	12	3	17	87
Changes in small bowel evacuation	17	8	4	4	2	10	45
Small bowel bleeding or remote hemorrhage	6	1	2	21	1	2	33
Yellow spots	9	3	3	5	2	9	31
Polyp of small intestine	6	2	3	4	0	7	22
Small bowel mass	5	0	0	2	0	8	15
Diverticulum of small intestine	4	1	0	0	0	3	8
Absence of villi	3	0	2	0	0	0	5
Vasculopathy	3	0	0	1	0	0	4
Pale intestinal mucosa	0	0	0	0	0	1	1
Small bowel erythema	1	0	0	0	0	0	1
Summary	406	124	113	84	35	212	974

*MCRCE, magnetically controlled robotic capsule endoscopy; GIB, gastrointestinal bleeding*.

## Discussion

The anatomical structure of the small intestine is so complex that conventional endoscopy cannot fully examine the entire small intestine. Enteroscopy is a commonly used procedure for diagnosing small bowel diseases. As an invasive examination, it requires a high degree of technical operating skills and a long operating time for physicians. Moreover, enteroscopy is associated with perforation, pancreatitis, and bleeding (8, 9). In contrast, small bowel angiography is used to identify and qualitatively diagnose unexplained gastrointestinal bleeding; however, the diagnostic accuracy of angiography is determined by the level of intubation, bleeding, bowel movements, and other factors. The diagnostic results of angiography are often negative in the bloodless state, which can limit its clinical application (5, 10).

In 2014, we began to use MCRCE to detect small intestinal disease, providing a non-invasive examination of the whole small intestine. It overcame the restriction of conducting endoscopy for patients with history of severe cardiopulmonary or spinal diseases, and endoscopy is known to be contraindicated in these cases. MCRCE can provide patients, who have absolute or relative traditional endoscopic contraindications, with timely endoscopy, early diagnosis, and treatment. MCRCE not only provides painless and non-invasive examinations, but also prevents cross-infection, narcotic drug risk, and other adverse effects in patients. All these factors make MCRCE a safer and more acceptable examination technology for small bowels (11).

In this study, the majority of patients successfully completed the examination. Six of the subjects were lost to follow up, and 158 of the subjects were excluded due to inadequate etiological data (Figure 1A). Related studies have shown that capsule retention can be ascribed to small intestinal tumors and inflammatory bowel disease, with an incidence of approximately 2% (12, 13). In this study, none of the capsules were retained in the bowels. Prior to the endoscopic examination, patients were examined by abdominal CT to confirm no intestinal stenosis or obstruction. Although the incidence of Crohn's disease among the patients enrolled in the study was probably minimal, strict control of indications for MCRCE can effectively reduce the incidence of complications.

Further, MCRCE helped detect small intestinal lesions in 974 of the 1,802 patients with gastrointestinal symptoms. Therefore, the positive detection rate of 54.1% suggests a high incidence of small bowel disease in patients with chronic abdominal pain, abdominal distension, diarrhea, and unknown bleeding. MCRCE is highly clinically valuable in the detection of small intestinal diseases, consistent with the findings of previous studies (3, 14, 15). In the 974 positive cases, the incidence of non-specific enteritis (722 cases, accounting for 40.1%) was the highest, and the positive rate was higher than the detection rate of ordinary capsule endoscopy (16), suggesting that chronic abdominal pain, abdominal distension, and diarrhea are associated with poor colon function and mild changes in the physiological structure of the small intestine. Based on clinical experience, glutamin entersoluble capsules or probiotics, including Bifidobacterium (17), were used in patients with non-specific enteritis. As for patients with suspected inflammatory bowel disease, we will use other inspections (CT enterography, magnetic resonance imaging enterography, balloon-assisted enteroscopy) to further identify the diagnosis and treatment of the disease.

Small intestinal bleeding emerges from between the suspensory ligament of the duodenum and the Bauhin's valve, accounting for 5% of all gastrointestinal bleeding (GIB) and 80% of obscure GIB cases (18). Common causes of small intestinal bleeding include cancer, vascular malformations, and inflammatory bowel diseases (19). In this study, 84 of 149 cases of unexplained GIB were observed to have positive lesions in the small bowel, with a detection rate of 56.4% (84/149). The main causes were non-specific enteritis and ulcer of the small intestine (Tables 1, 2).

However, the main limitations of MCRCE include: (i) different observed areas depending on the movement trajectory of the capsule; (ii) the long time of processing and reporting (20); (iii) absence of biopsy; (iv) the effect of bowel preparation on the diagnostic yield (21). Moreover, the potential diagnostic yield of MCRCE was low in this study due to the quite generic inclusion criteria, which requires improvement in further studies.

This study suggests that MCRCE is a potential primary choice for the examination of small bowel diseases. With the progress of science and technology, capsule endoscopes with more comprehensive functions are being developed, such as biopsy capsules, hemostatic capsules, and drug transport capsules (22–24). We believe that these capsules, being more flexible and controllable, which can be used for biopsy or treatment, will have a significant value for clinical applications in the future.

## Conclusion

MCRCE is a safe and non-invasive technology, with a highly accurate detection rate in diagnosing small intestine diseases, which is significantly valuable for clinical application in patients with suspected small intestine diseases.

## Data Availability Statement

The raw data supporting the conclusions of this article will be made available by the authors, without undue reservation.

## Ethics Statement

The studies involving human participants were reviewed and approved by the clinical research was approved by the medical device ethics committee of Shanghai Jiao Tong University Affiliated Sixth People's Hospital. The patients/participants provided their written informed consent to participate in this study.

## Author Contributions

J-SZ and JZ supervised the study. X-YC and JZ oversaw the study design. X-YC, WD, RL, H-NF, Y-CY, MC, and H-WQ performed the procedures. X-YC, WD, and JZ performed the data analysis. X-YC prepared the manuscript while JZ and J-SZ revised the manuscript. All authors contributed to the article and approved the submitted version.

## Conflict of Interest

The authors declare that the research was conducted in the absence of any commercial or financial relationships that could be construed as a potential conflict of interest.
